# Bridging data gaps and tackling human vulnerabilities in healthcare cybersecurity with generative AI

**DOI:** 10.1371/journal.pdig.0001063

**Published:** 2025-10-28

**Authors:** Mohammad S. Jalali, Karen Largman

**Affiliations:** MGH Institute for Technology Assessment, Harvard Medical School, Boston, Massachusetts, United States of America; CHOP: The Children's Hospital of Philadelphia, UNITED STATES OF AMERICA

## Introduction

Cybersecurity is an ever-evolving challenge across industries, but the stakes are particularly high in healthcare. The sector’s reliance on interconnected systems, including electronic health records, emerging medical technologies, and AI-driven diagnostic tools, combined with insufficient resources to build robust cybersecurity capabilities, makes it a prime target for cyberattacks [1].

Within healthcare, hospitals are especially vulnerable. They have increasingly faced targeted cyberattacks that exploit both technological and human vulnerabilities. For example, in 2021, a ransomware attack paralyzed Ireland’s national healthcare system, canceling thousands of appointments and delaying patient care [2]. Phishing attacks remain a leading cybersecurity threat in U.S. healthcare organizations. A 2023 survey found that nearly 59% of major security incidents started with general email phishing, with additional cases linked to spear-phishing (targeted phishing attacks) and SMS phishing [3]. These attacks often impersonate internal communications like Electronic Health Records (EHR) updates, billing notices, or payroll alerts to steal staff credentials and access sensitive patient information. While these examples highlight specific incidents, human decision-making is the common thread that determines how incidents arise, escalate, and are managed.

Unlike industries such as finance, which employ centralized systems and end-to-end encryption to safeguard customer data, healthcare’s outdated legacy systems and fragmented IT infrastructures exacerbate its vulnerability. These structural weaknesses, compounded by stringent privacy regulations and logistical barriers, further hinder the collection and sharing of data needed for evidence-based research and innovation in cybersecurity [1,4]. While some data like individual and network activity logs are often collected, privacy concerns and regulatory restrictions frequently prevent their meaningful use, such as analyzing patterns to identify vulnerabilities or developing tailored interventions.

Moreover, human behavior is a particularly significant factor in healthcare cybersecurity, as most health record breaches stem from poor security practices. For example, phishing attacks in hospitals frequently mimic legitimate administrative requests, such as updates from the payroll department, EHR system maintenance alerts, or patient billing communications. Administrative staff may be targeted with requests that appear operationally routine. Clinical staff, often under time pressure and navigating patient care demands, may overlook subtle inconsistencies in these messages, especially under high workload, which has been shown to significantly increase the likelihood of clicking on phishing links [5].

Importantly, these vulnerabilities often arise not from individual negligence but from the misalignment between security protocols and clinical workflows. Burdensome authentication processes, poorly integrated security tools, and fragmented systems can push staff toward insecure workarounds. Decades of research emphasize user-centered security design, yet systematic data on workflow-security interactions remain scarce; technology metrics are still collected far more often than behavioral, cultural, or process metrics [6,7]. This imbalance leaves a blind spot in understanding human actions and decision-making vulnerabilities.

Simulation modeling, such as agent-based models and Markov decision processes, has long been used to explore human behavior in complex systems. In cybersecurity, such models offer a way to test intervention strategies, account for workflow constraints, and simulate decision-making under stress. However, these approaches have been limited by the lack of detailed, representative behavioral data, particularly in healthcare settings. This data scarcity has hindered the realism and usefulness of such simulations.

Leveraging generative AI offers a promising approach to addressing critical data gaps by incorporating its outputs in simulation models that represent human decision-making and behavioral responses. For example, a model could generate synthetic staff responses to phishing attempts under different roles and time pressures and integrate these data into simulation models to test interventions. Such simulations could be validated using limited real-world behavioral data, expert input, and benchmarking, including small-scale pilot testing of scenarios with clinical and administrative staff, to ensure the generated data realistically capture hospital workflows. Although generative AI is still evolving and its application to healthcare cybersecurity is in its infancy, its potential to uncover complex challenges warrants exploration and careful evaluation.

This paper offers a conceptual perspective rather than a deployment-ready solution. Our goal is to highlight an emerging research direction that addresses vulnerabilities in healthcare, particularly hospital cybersecurity, by using synthetic behavioral data. While synthetic patient populations or clinical data are generated to test interventions in healthcare modeling, similar methods need to be adapted for cybersecurity analysis; for example, by modeling human decision-making under uncertainty with simulated users and adversaries.

## Bridging data gaps in cybersecurity’s human dimension

Recent research highlights the potential of synthetic data to address technological challenges, such as improving anomaly detection, identifying threats from the Internet of Things, and testing system robustness [8–10]. However, the application of generative AI to model human behavior in cybersecurity remains largely underexplored. Decision-making behaviors in hospital settings, such as how staff balance security protocols with clinical urgency, often follow structured patterns shaped by role, task type, and institutional context. These patterns, while variable, create plausible decision spaces for modeling vulnerabilities and intervention strategies.

Unlike approaches focused on detecting malicious traffic, we propose generating synthetic behavioral data to explore human decision-making vulnerabilities in hospital cybersecurity. This approach could simulate plausible staff responses to security threats, such as phishing attempts, credential sharing under pressure, or adherence to security protocols during high-stress periods. For example, a generated dataset could simulate staff reactions before and after phased multi-factor authentication rollouts or revised password policies, allowing researchers to test how such interventions might affect decision-making and workflow under different roles and time pressures.

If comprehensive real-world datasets of hospital cybersecurity behaviors were available, the need for synthetic generation would be reduced. Because of the persistent sensitivity, privacy, and complexity of such data, we may never fully capture them, making generative approaches a pragmatic tool for approximating behavioral patterns. Given these persistent gaps, synthetic approaches offer a practical and ethical alternative for exploratory modeling.

While the potential is promising, achieving these outcomes requires substantial research and consideration of major challenges, ensuring that generated behavioral data are representative of real decision-making pressures and realistically capture the hospital ecosystem. If they fail to capture the nuances of complex human behavior—such as how staff balance efficiency and security, or make rare but critical outlier decisions during security incidents—the resulting interventions may lack practical relevance.

Capturing subgroup-specific variations is especially critical in hospitals, where clinical staff, IT teams, and administrative personnel operate under distinct workflows and time pressures. For instance, night-shift nurses prioritizing rapid emergency access may face different cybersecurity trade-offs than daytime administrative staff handling billing systems. In practice, this differentiation can be built into the generative process by supplying role- and context-specific parameters, such as staff type, workload intensity, shift timing, and access urgency, as conditioning variables. Distinct synthetic decision patterns can be generated for each subgroup, which can be benchmarked against limited real-world observations or expert input to check plausibility before being used in simulation models.

Most generative AI models like GPT-5 are already pre-trained on very large general-purpose datasets. These pre-trained systems would typically be guided or conditioned with limited domain-specific behavioral data and expert input rather than trained from scratch. For those wishing to build their own models, smaller generative systems could be trained on privacy-preserving behavioral logs, simulated phishing exercises, or blended synthetic datasets, but this would require substantial resources and strict governance.

While these approaches make it feasible to explore human decision-making vulnerabilities, they also introduce new risks. Data generated through AI-based technologies can suffer from hallucination, where the models produce inaccurate or nonsensical data that may undermine the validity of analyses. Moreover, biases in generated data can emerge from incomplete or skewed assumptions. For example, oversimplifying staff responses to phishing attempts might result in training programs that fail to address vulnerabilities in specific subgroups, such as administrative staff versus clinical providers.

Finally, while generative AI technologies offer potential, their use also raises cybersecurity concerns. Specifically, data inputs used to train or prompt large language models could inadvertently expose confidential information. To mitigate this risk, organizations must anonymize inputs, restrict sensitive data exposure, and use large language models in secure environments.

## Toward a balanced approach

To fully harness the potential of generated data, several steps are necessary. First, generated data must be tailored to the specific context and challenges of healthcare cybersecurity, requiring close collaboration with domain experts to ensure a realistic representation of system and staff behavior. In hospitals, cybersecurity measures often clash with the realities of clinical urgency. For example, lengthy multi-factor authentication can delay care, prompting staff to share credentials or leave workstations unlocked. To understand these challenges, behavioral researchers, data scientists, IT and security experts, and healthcare professionals should work together to generate data that are reliable and behaviorally relevant. Such collaborative efforts can also support the development of decision-making simulations, exploring vulnerabilities and potential interventions to improve real-world applicability, while minimizing staff burden due to existing workload and time pressure. Continuous iteration between generated data, real-world data, expert feedback, and simulation outcomes is critical to refining simulation models and improving their predictive accuracy. Implementing these collaborative and iterative improvements also requires significant resources, which can be a barrier for underfunded healthcare organizations.

Second, validation frameworks must be developed to assess the realism, fidelity, and applicability of generated datasets. For instance, a benchmarking system could assess how effectively generated data captures the dynamics of human behavior, such as decision-making when confronted with a phishing email. Third, ethical safeguards must address privacy concerns, mitigate biases in both data generation and application, and ensure responsible use by establishing clear guidelines and monitoring mechanisms to prevent misuse. These safeguards should also address the risks of generated data being repurposed for malicious activities.

Finally, the limitations of generated data must also be acknowledged. Generated data cannot fully replace real-world data, as certain emergent behaviors, such as team-based responses during coordinated phishing attacks or contextual decision-making under emergency conditions, may only arise in live hospital environments. Careful dataset construction and grounding in evidence are necessary to avoid biases or misleading conclusions. Generated data should also complement, not substitute, efforts to improve real-world data collection and sharing.

Integrating simulation modeling with generated data, improved real-world data practices, and multidisciplinary collaboration can help build resilient cybersecurity strategies. This integrated approach helps overcome longstanding behavioral data limitations and enhances current cybersecurity capabilities.
